# 3D Hierarchical Polyaniline–Metal Hybrid Nanopillars: Morphological Control and Its Antibacterial Application

**DOI:** 10.3390/nano11102716

**Published:** 2021-10-14

**Authors:** Jueun Kim, Younseong Song, Hogi Kim, Nam-Ho Bae, Tae Jae Lee, Yoo Min Park, Seok Jae Lee, Sung Gap Im, Bong Gill Choi, Kyoung G. Lee

**Affiliations:** 1 Center for Nano Bio Development, National NanoFab Center (NNFC), 291 Daehak-ro, Yuseong-gu, Daejeon 34141, Korea; jekim@nnfc.re.kr (J.K.); nhbae@nnfc.re.kr (N.-H.B.); tjlee@nnfc.re.kr (T.J.L.); ympark@nnfc.re.kr (Y.M.P.); sjlee@nnfc.re.kr (S.J.L.); 2 Department of Chemical and Biomolecular Engineering, Korea Advanced Institute of Science and Technology (KAIST), 291 Daehak-ro, Daejeon 34141, Korea; ong12ong12@kaist.ac.kr (Y.S.); hokiepokie91@kaist.ac.kr (H.K.); sgim@kaist.ac.kr (S.G.I.); 3 Department of Chemical Engineering, Kangwon National University, Samcheok 25913, Korea

**Keywords:** hierarchical structure, polyaniline–metal hybrid, antibacterial application

## Abstract

Effective and reliable antibacterial surfaces are in high demand in modern society. Although recent works have shown excellent antibacterial performance by combining unique hierarchical nanotopological structures with functional polymer coating, determining the antibacterial performance arising from morphological changes is necessary. In this work, three-dimensional (3D) hierarchical polyaniline–gold (PANI/Au) hybrid nanopillars were successfully fabricated via chemical polymerization (i.e., dilute method). The morphology and structures of the PANI/Au nanopillars were controlled by the reaction time (10 min to 60 h) and the molar concentrations of the monomer (0.01, 0.1, and 1 M aniline), oxidant (0.002, 0.0067, 0.01, and 0.02 M ammonium persulfate), and acid (0.01, 0.1, 1, and 2 M perchloric acid). These complex combinations allow controlling the hierarchical micro- to nanostructure of PANI on a nanopillar array (NPA). Furthermore, the surface of the 3D PANI/Au hierarchical nanostructure can be chemically treated while maintaining the structure using initiated chemical vapor deposition. Moreover, the excellent antibacterial performance of the 3D PANI/Au hierarchical nanostructure (HNS) exceeds 99% after functional polymer coating. The excellent antibacterial performance of the obtained 3D PANI/Au HNS is mainly because of the complex topological and physicochemical surface modification. Thus, these 3D PANI/Au hierarchical nanostructures are promising high-performance antibacterial materials.

## 1. Introduction

Antimicrobial resistance in infectious microorganisms, especially in pathogenic bacteria, is a major global health concern [1]. Researchers expect the risk of antibacterial resistance to reach 10 million by 2050 [2]. In nature, continuous bacterial colonization on surfaces could adversely affect the function of various interfaces, such as those on surgical tools, walls, aquatic flow systems, daily supplies, textiles, contact lenses, and medical implants [3,4]. In particular, pathogenic bacterial attachment, proliferation, and colonization on surfaces could result in the rapid spread of infectious bacteria [5,6]. Therefore, substantial exploration of antimicrobial treatment approaches that involve inhibiting and/or eliminating initial bacterial attachment is of special interest to prevent the transmission of pathogens.

To date, metal nanoparticles composed of zinc, titanium, carbon, and copper have been successfully employed in antibacterial applications, offering infection control and management of biofilm formation [7,8,9]. However, these metal particles may cause severe cytotoxicity and long-term stability issues. Apart from using nanoparticles, coating surfaces with antibiotic releasement materials, such as peptide and quaternary ammonium compounds, has become an alternative to developing an antibacterial surface [10,11]. More recently, numerous studies have focused on developing advanced antibacterial performance by adopting inorganic nanostructures. For example, Pogodin et al. and Bandara et al. reported an excellent bactericidal effect by mimicking the nanostructures of insect wings [12,13]. In addition, a titanium oxide nanopillar array (NPA) was developed to confirm its advanced bactericidal activity and develop an understanding of the mechanistic processes that lead to bacterial death via penetration of the bacterial cellular membrane by NPAs [14]. Although superior antibacterial performance of inorganic-based nanostructures has been verified, their high stiffness and inflexibility make them more vulnerable to shear forces and limit the expansion of their antibacterial applicability [15,16].

To overcome these problems, alternative approaches have been developed to improve the fabrication of 3D hierarchical organic–inorganic hybrid structures [17,18,19]. Sasidharan et al. reported 3D polymer–metal–carbon hybrid materials to remove biological contaminants and provide antibacterial performance [20]. In addition, coating or implantation of nanomaterials, including zinc and copper oxide, on polymers has demonstrated excellent reliability and antibacterial performance [15,21,22]. In addition, our group has studied how pathogenic bacteria interact with 3D hierarchical nanostructures and their capability for early-stage detection of pathogens and antibacterial performance [23]. Although these studies provide valuable information, the effect of the molar concentrations of monomer, oxidant, and acid in polyaniline (PANI)-based nanostructures and their effect on antibacterial performance are not well understood. Studying the morphologies of 3D hierarchical nanostructures arising from the effects of reaction conditions during the polymerization process is necessary. Moreover, the study of morphological control; the excellent flexibility, elasticity, and biocompatibility from organic materials; and the mechanical stability and resilience from inorganic materials enable achieving reliable and cost-competitive fabrication of antibacterial hybrid composite materials.

In this study, we examined the effect of concentration and acidity on the PANI-based nanotopography and antibacterial performance. Submicrometer NPAs were used as the main substrate to grow PANI nanofibers. The elastic NPA was prepared via photolithography using a master mold and etching of a silicon wafer, and the NPA was replicated using a UV-curable polymer blend of polyurethane acrylate (PUA) and NOA63, called PUNO [15,24]. Further, the antibacterial capability PANI-modified NPAs was chemically modified using a copolymer of 4-vinylbenzyl chloride (VBC) and 2-(dimethylamino)ethyl methacrylate (DMAEMA) (p(VBC-co-DMAEMA) or pVD) via initiated chemical vapor deposition (iCVD) [25,26,27]. To verify the antibacterial performance of the as-prepared PANI nanostructures on NPAs, *Escherichia coli* (*E. coli*) O157:H7 was selected as a model bacterium because it is the most well-known pathogen that causes foodborne illness in daily life. Controlled molar concentrations of aniline, oxidants, and acidity were used as variables to understand the morphological changes in PANI and antibacterial performance.

## 2. Materials and Methods

### 2.1. Fabrication of a Si Master Mold

The Si nanohole mold was prepared according to a previously reported procedure by our group. The Si wafer was placed in a furnace (Furnace E1200, Centrotherm, Blaubeuren, Germany) to form a 500 nm thick SiO_2_ layer. The thermally oxidized wafer was spin-coated with 0.7 μm of photoresist; then, 500 nm dots were patterned using a KrF scanner (S203-B, Nikon, Tokyo, Japan). Following these steps, the Si wafer was etched using ICP (TCP9400SE, Lam Research, CA, USA) with gas mixtures including Cl_2_, HBr, and O_2_ to produce Si nanoholes. Finally, the nanohole array was patterned on the Si wafer, which was used as a mold to fabricate the NPAs.

### 2.2. Preparation of a PANI Nanostructure

**Figure 1:**
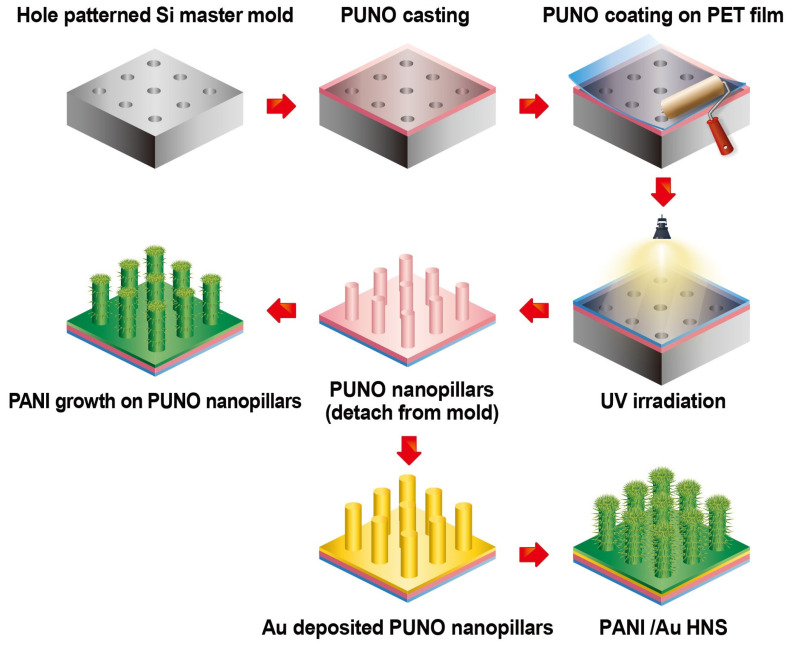
Schematic of the fabrication of the PANI nanostructure.

Prior to synthesizing PANI, the PUNO NPA on polyethylene terephthalate (PET) film was prepared by a combined photo- and soft lithography technique using a Si nanohole master mold. A mixture of Norland Optical Adhesive 63 and PUA (MINS-311RM, Minuta Tech., Chungcheongbuk-do, Korea) was coated on the surface of the master mold using a spin coater (LSM250, SAWATEC, Jiangsu, China). After exposure to UV (EVG6200, EVG, DI Erich Thallner Strasse, Austria) for 1 min, the free-standing PUNO NPA film was peeled from the master mold. To provide a current collector, a thin conformal layer of gold/titanium was deposited onto the NPAs via vacuum evaporation. The PANI was synthesized on the surface of PUNO film via dilute polymerization. The PUNO film was immersed in a mixture solution of perchloric acid (60%, JUNSEI, Tokyo, Japan), APS (98%, Sigma-Aldrich, Seoul, Korea), aniline (99.5%, Sigma-Aldrich, Seoul, Korea), and deionized water. The molar concentrations of aniline monomer, perchloric acid, and APS varied in the range of 1‒1000 mM to control the PANI morphology (Figure 1). Polymerization was performed at 4 °C and the product incubated for 10 min to 60 h. The PANI/Au HNS was obtained after thoroughly washing with ethanol and deionized water several times.

### 2.3. Antibacterial Coating

An antibacterial thin film of a copolymer of 4-vinylbenzyl chloride (VBC) and 2-(dimethylamino)ethyl methacrylate (DMAEMA) (p(VBC-co-DMAEMA) or pVD) was deposited on the PANI nanostructure via iCVD. The deposition was performed in the same conditions as those in the previous study. In particular, DMAEMA and VBC monomers and an initiator of tert-butyl peroxide (TBPO) were kept at 50 °C, 40 °C, and room temperature to vaporize into the iCVD chamber; their corresponding flow rates were 1.729, 0.406, and 0.567 sccm. The chamber was maintained at 250 mTorr, and the substrate temperature was kept at 30 °C. The reaction was initiated by heating the filament to a temperature of 140 °C. The thickness of the thin film on a wafer was monitored using an M-2000 VI Spectroscopic Ellipsometer (J.A. Woolam Co., Inc., Lincoln, NE, USA).

### 2.4. Preparation of an Antibacterial Test

Bactericidal experiments were performed on the typical etiological bacteria, Gram-negative *E. coli* O157:H7 (ATCC 43894), provided by the Korea Research Institute of Bioscience and Biotechnology (Daejeon, Korea). The Luria–Bertani (LB) broth was fabricated with the addition of sodium chloride, yeast extract, and tryptone to 100 mL of distilled water kept at 37 °C for 18 h. The bacteria were cultured in the LB broth to obtain 1.0 × 10^8^ CFU/mL. In a typical experiment, 1 mL of *E. coli* suspension (1.08 × 10^4^ CFU/mL) was spotted on the surface of samples, including pristine cell culture dish, pVD-coated cell culture dish, PANI film, and pVD-coated PANI film with a 35 mm diameter. The suspension was recovered and serially diluted to be spread on LB agar solid plates for bacterial enumeration. After incubation for 16 h, the colony count ranged from 30 to 300 CFU.

### 2.5. Statistical Analysis

The antibacterial efficiencies, performed in triplicate, were expressed as the mean values ± standard deviation. Statistical analysis was performed using one-way ANOVA, where *p* < 0.05 was considered statistically significant (* *p* ≤ 0.05, ** *p* < 0.01, and *** *p* ≤ 0.001).

### 2.6. Characterizations

Field-emission scanning electron microscope (SEM, Hitachi S-4800, Chungbuk, Korea) was performed to investigate the morphology of the PUNO NPA and the 3D hierarchical structure of PANI/Au. Fourier-transform infrared (FTIR) spectroscopy spectra were recorded using FTIR-4600 (Jasco, Tokyo, Japan). Each spectrum was recorded from 4000 to 300/cm.

## 3. Results

### 3.1. Subsection Effect of Reaction Time on PANI Morphology

To obtain highly ordered NPAs, a polymer blend of PUA and NOA63 was spin-coated onto a prefabricated silicon mold, followed by UV curing and removal by peeling from the mold (Figure S1) [28]. The NPAs have a diameter, height, and center-to-center distance of 500 nm, 1.25 μm, and 1 μm, respectively; these dimensions are easily replicated and produced using a single silicon wafer mold (8-inch diameter). The schematic of the fabrication procedure of the PANI/Au hierarchical nanostructure (HNS) is shown in Figure 1. The highly nanopillar arrayed polymer film was initially fabricated by a combination of photo- and soft lithography. In addition, 20 nm thick titanium (Ti) and 200 nm thick gold (Au) layers were deposited on the surface of the NPA film to extend the growth of PANI. To synthesize a unique 3D hierarchical structure based on NPAs, the dilute polymerization method enabled the degree of PANI nanostructure growth to be controlled [28,29,30]. In general, chemical polymerization for PANI synthesis occurs when a pH of 3 or less and strong oxidants are applied; the lower the pH, the lower is the probability of the formation of a branched polymer structure [31,32,33]. Based on these mechanisms, the effects of controlling reaction time, molar concentration of monomer, molar concentration of oxidant, and acidity are determined by the morphology of the PANI.

**Figure 2:**
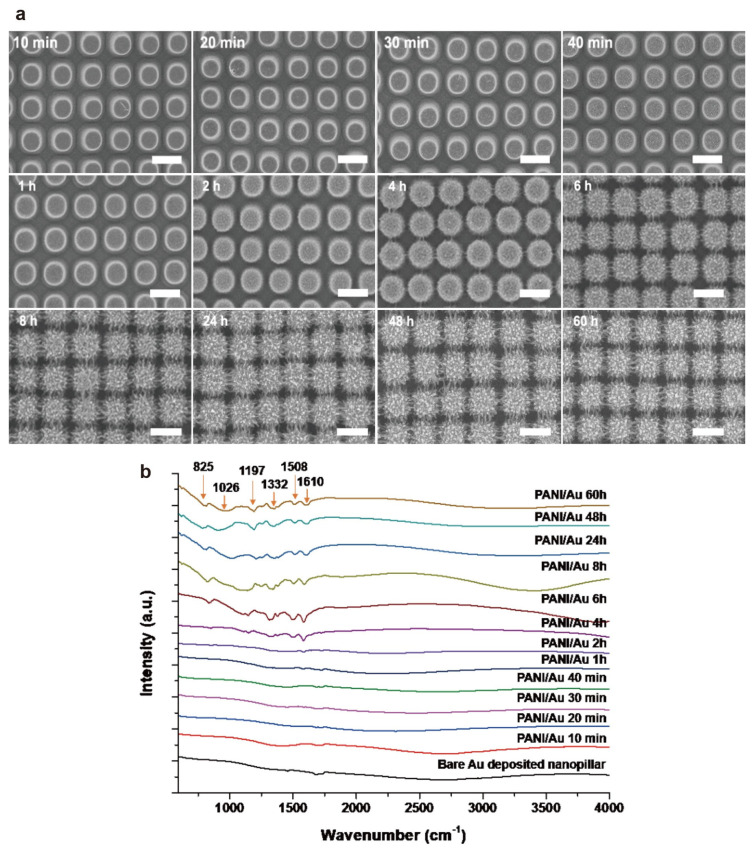
(**a**) SEM images and (**b**) FTIR spectra of the PANI nanostructure under different reaction times, from 10 min to 60 h. The scale bar is 1 μm.

To investigate and understand PANI nanofiber construction on NPAs, the morphological and chemical structural changes were observed via scanning electron microscopy (SEM) and Fourier-transform infrared (FTIR) spectroscopy. Figure 2a shows the SEM images of the PANI nanofibers on Au-deposited NPAs. The reaction was performed under different reaction times, ranging from 10 min to 60 h, using 0.01, 0.0067, and 1 M of aniline, ammonium persulfate, and perchloric acid, respectively. During the reaction time from 10 min to 1 h, no significant morphological changes were observed in the SEM images. Interestingly, after 2 h of PANI growth, numerous small dots slowly appeared on the NPA surface. In addition, the SEM images (Figure 2a) show the formation of a PANI nanofiber bridge at 4 h and the gradual growth of spike-shaped PANI fibers after 6 h of reaction. Moreover, the highly dense PANI nanofibers and networks were successfully constructed around the NPA after 8 h of reaction, but almost no significant morphological changes were observed even after increasing the reaction time to 60 h. Moreover, the nanofibers have different diameters and lengths, ranging from 50 to 80 nm and from 150 to 250 nm, respectively. Notably, the PANI nanofiber grew after 6 h of reaction time and was connected to the PANI nanofiber grown in the adjacent NPAs, resulting in a network structure. The area of these network structures gradually increased and formed a PANI/Au HNS.

In addition to the physical morphological changes, chemical changes were observed as a function of PANI reaction time from 10 to 60 h using FTIR (Figure 2b). During PANI growth on the NPAs, almost no signature peaks were observed up to 1 h of the PANI reaction. Interestingly, the unique adsorption peaks at 1508 and 1610 cm^−1^ appeared at 2 h of reaction time, indicating PANI nanofiber formation. The adsorption peaks at 825 and 1026 cm^−1^ correspond to the out-of-plane C–H and in-plane C–H bending modes, respectively. The peak around 1197 cm^−1^ indicates the conducting protonated form, as this feature is known to arise from the C–N^+^ stretching mode in the polaron form of PANI. The peaks at 1508 and 1610 cm^−1^ can be attributed to the C=C and C=N stretching modes of the benzenoid and quinoid groups of the PANI, respectively. The peak at 1332 cm^−1^ can be assigned to a C–N stretching mode of the benzenoid unit. The absorbance band at 2930 cm^−1^ is that of an aromatic C–H stretch, whereas the peak at 3280 cm^−1^ corresponds to the N–H stretching mode of an aromatic amine. Most of the distinctive peaks at 825, 1026, 1197, 1332, 1508, and 1610 cm^−1^ exhibited and confirmed the successful synthesis of PANI nanofibers [34,35,36]. Furthermore, both the morphological and chemical formation of PANI were fairly matched and occurred after 2 h of reaction.

### 3.2. Effect of Reagents on PANI Structures

**Figure 3:**
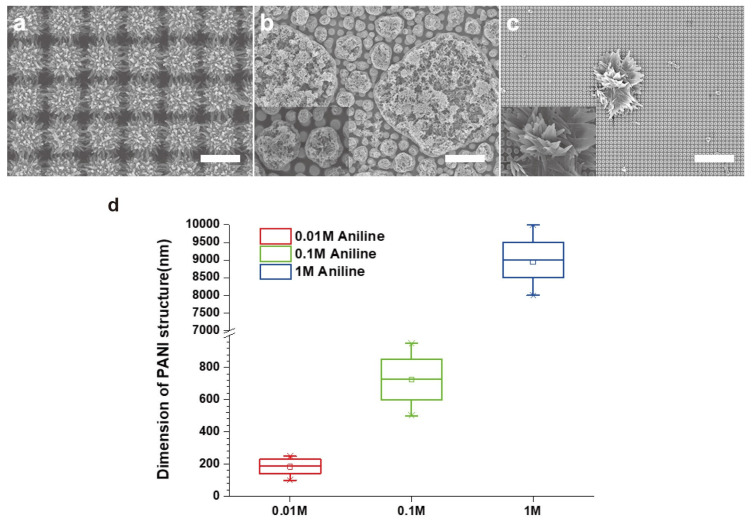
SEM images of PANI synthesized with different aniline concentrations: (**a**) 0.01 M, (**b**) 0.1 M, and (**c**) 1 M. Scale bars are 1 μm, 4 μm, and 10 μm, respectively. (**d**) Diagram of PANI dimensions.

The monomer concentration is another important parameter affecting the PANI morphology. To understand the effect of monomer concentration, PANI was synthesized under different molar concentrations of aniline: 0.01, 0.1, and 1 M. At a concentration of 0.01 M, PANI was fully covered and formed a nanofiber network on the NPA surface as observed in the SEM image (Figure 3a). At 0.1 M, PANI grew rapidly in the reactant to form numerous micrometer-scale clusters on the NPA but no PANI nanofibers and/or nanonetworks were fabricated (Figure 3b). As the molar concentration of aniline increased to 1 M, macroscale flower-like structures grew on the NPA (Figure 3b,c). The relatively low molar concentration of aniline resulted in more acidic conditions and easily formed numerous seeds. These seeds were immobilized on the surface of the gold to construct fibrous structures on the NPA surface. In contrast, as the molar concentration of aniline increased, the solution became more alkaline and fewer seeds were produced. In addition, the slow chemical polymerization of PANI produced microscale clusters and/or flower-like structures on the NPA surface [34,37,38,39,40].

**Figure 4:**
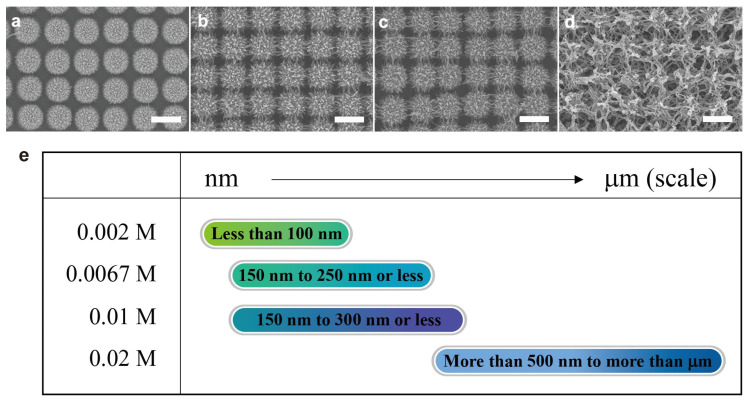
SEM images of PANI synthesized with different ammonium persulfate concentrations: (**a**) 0.002 M, (**b**) 0.0067 M, (**c**) 0.01 M, and (**d**) 0.02 M. The scale bar is 1 μm. (**e**) Diagram of APS concentration vs. tendency of PANI structures (length).

To investigate the effect of the concentration of ammonium persulfate (APS), concentrations of 0.002, 0.067, 0.01, and 0.02 M of the oxidant were applied while maintaining the molar concentration of the other reagents. SEM images of synthesis of PANI on the surface of the Au-deposited NPAs are shown in Figure 4. Figure 4a shows the SEM image at an oxidant concentration of 0.002 M. The PANI nanostructures grew on the NPA surface but were partially covered by a spiked shape (Figure 4a). At 0.0067 M and 0.01 M, PANI nanofibers clearly connected with each other to form a nanonetwork (Figure 4b,c). However, at a molar concentration of 0.02 M, the reaction-rate-determining step of PANI synthesis shortened and the germination occurred faster, resulting in the formation of a denser nanonetwork and overgrowth on the surface of the NPA (Figure 4d). The series of SEM images indicate that the oxidant of APS can determine the nanostructural shape of PANI.

**Figure 5:**
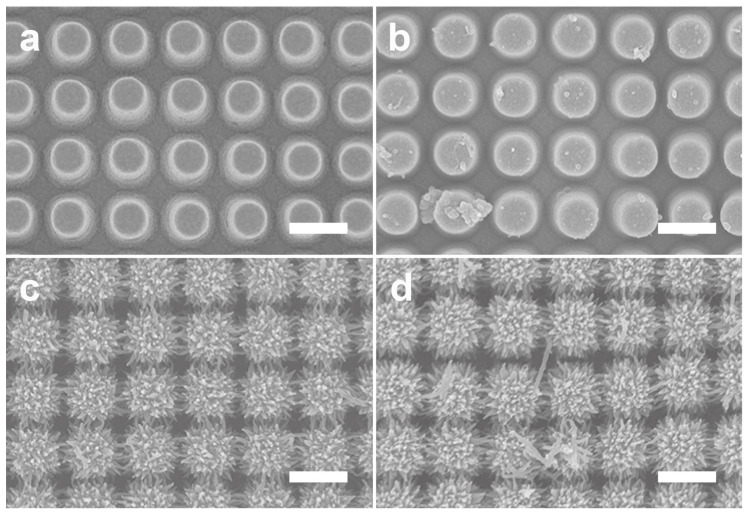
SEM images of PANI synthesized with different perchloric acid concentrations: (**a**) 0.01 M, (**b**) 0.1 M, (**c**) 1 M, and (**d**) 2 M. The scale bars is 1 μm.

Modulation of acidity is another important parameter to control the nanostructural shape of PANI. To control the acidity, which is a major requirement for PANI polymerization, we applied 0.01 M (pH 3.99), 0.1 M (pH 3.00), 1 M (pH 1.99), and 2 M (pH 1.69) of perchloric acid to synthesize PANI on the NPA surface. Interestingly, in conditions below 0.1 M (≥pH 3) of perchloric acid, there were no visible PANI nanofibers (Figure 5a,b). However, in strong acidic conditions (below pH 2), PANI nanofibers were successfully synthesized and formed networks (Figure 5c,d). According to previous reports, PANI nanostructures, nanofibers, or nanotubes can be fabricated under acidic conditions with pH less than 2.5 [40]. In addition, the planar two-dimensional nanostructure forms predominantly over the one-dimensional nanostructure. Therefore, high acidity is an essential parameter to fabricate PANI nanostructures and its optimal concentration for this structure is approximately 1 M of perchloric acid.

### 3.3. Synthesis Mechanism of PANI

**Figure 6:**
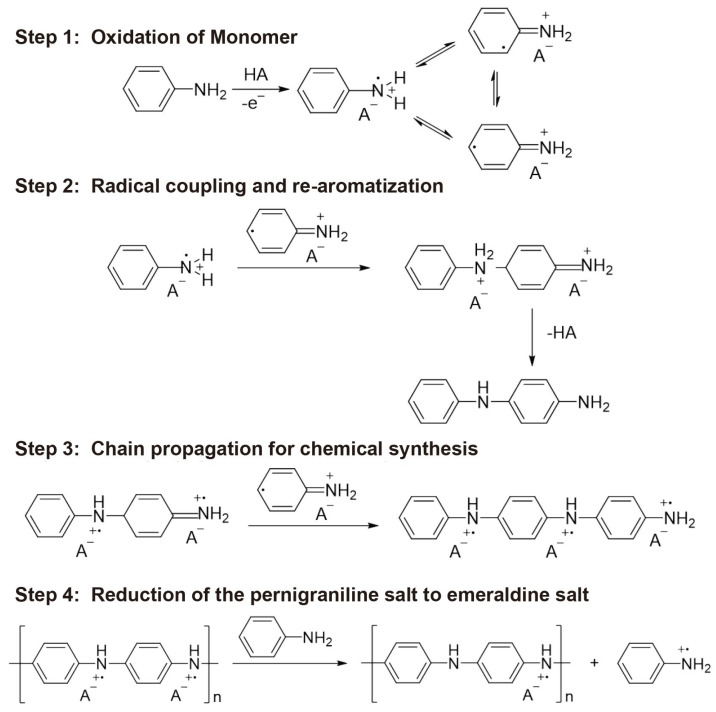
Polymerization mechanism of PANI.

The chemical polymerization of aniline proceeds via a radical propagation mechanism comprising the following four steps: (1) oxidation of the monomer, (2) radical coupling and re-aromatization, (3) chain propagation for chemical synthesis, and (4) reduction of the pernigraniline salt to emeraldine salt (Figure 6). In step 1, the aniline monomer is oxidized to a radical cation; this is the slowest and therefore the rate-determining step in aniline polymerization. In step 2, the N-position and para-position subsequently produce a dicationic dimer, which further performs re-aromatization. These processes yield an intermediate form of p-aminodiphenylamine. In step 3, the dicationic dimer is oxidized either on the surface of the Au-coated NPA or in solution. The result of chain propagation from the dicationic dimer converts into the fully oxidized pernigraniline salt. In step 4, as the reaction mixture consumes all the oxidant, the pernigraniline salt formed in step 3 is reduced by unreacted aniline and produces green emeraldine salt [32,41]. Previous research indicates that the initial rate of aniline oxidation increases with the increasing initial pH of the reaction mixture.

When we increase the molar concentration of the oxidant, the mixture solution becomes more acidic (pH ≈ 1) and more suitable to form PANI nanofibers on the NPA, whereas more microscale clusters form in less acidic conditions (≥pH 3). Moreover, aniline is not completely oxidized at low oxidant concentrations. Based on the result shown in Figure 4, the effect of the oxidant clearly indicates that the dense nanonetwork can be formed by increasing the oxidant concentration. Furthermore, as the amount of perchloric acid was increased, more dense nanofibers and nanonetworks of PANI were observed, whereas no significant nanostructures were observed above pH 3 (Figure 5). Based on the obtained results, heterogeneous nano- to microscale PANI structures form on NPAs owing to the complex effect of the reaction time and molar concentrations of aniline, oxidant, and acid. Furthermore, the experimental results agree well with the proposed aniline polymerization mechanism (Table 1). Therefore, precisely controlling all of these parameters is important to synthesize the desired PANI structures on NPAs.

### 3.4. Antibacterial Test

**Figure 7:**
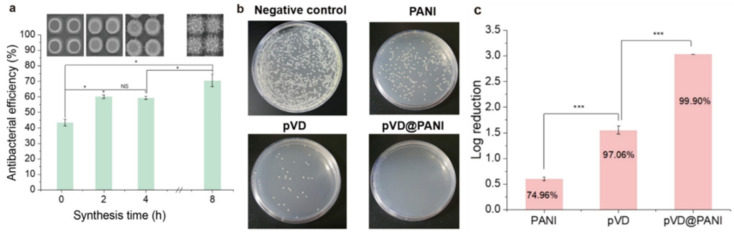
Antibacterial efficiency test using different types of hierarchical structures. (**a**) Antibacterial efficiency and corresponding SEM images (inset) of PANI nanofibers growing on an NPA with time. (**b**) Representative colony assays of antibacterial tests of a negative control, a PANI NPA, pVD thin film, and a pVD-coated PANI NPA. (**c**) Corresponding log reduction of bacteria with a PANI NPA, pVD thin film, and a pVD-coated PANI NPA. All data were obtained in triplicate. The significance was determined via one-way ANOVA analysis (* *p* ≤ 0.5, ** *p* < 0.1, and *** *p* < 0.01).

The role of hierarchical topography in and its effect on antibacterial performance are critical for application in various medical fields [42,43,44]. Using the submicrometer-scale hierarchical structure as an antibacterial substrate, the effect of hierarchical topography was investigated on the antibacterial performance against Gram-negative *E. coli* O157:H7. To this end, different types of hierarchical structures were fabricated under different reaction times, of 10 min, 2 h, 4 h, and 8 h, in which distinct structural changes occurred. As shown in Figure 7a, the antibacterial efficiency of the NPA after 10 min was 43.4% ± 2.16. After 2 h of reaction, the antibacterial efficiency of the NPA increased to 60.1% ± 1.16, whereas that of the PANI structure at 4 h exhibited no significant antibacterial performance changes. After PANI was grown for 8 h, its hierarchical structure enhanced the antibacterial performance up to 70.5% ± 3.95. Furthermore, the hierarchical structure enhanced the chemical interaction of antibacterial performance. To verify the synergetic effect of topography and chemical functionality, we adapted a thin ionic antibacterial polymer layer on the hierarchical structure via iCVD. In this case, a copolymer of 4-vinylbenzyl chloride (VBC) and 2-(dimethylamino)ethyl methacrylate (DMAEMA) (p(VBC-co-DMAEMA) or pVD) was selected as the antibacterial polymer because pVD shows excellent antibacterial performance using quaternary ammonium compounds [25]. For the colony assay, a pristine cell culture dish, a pVD-coated cell culture dish, and a pVD-coated PANI (pVD@PANI/Au HNS) structure with a size of 35 mm were incubated with a bacterial suspension (1.08 × 10^4^ CFU/mL) for 120 min and the bacterial proliferation was assessed by agar plate counting (Figure 7b). The pVD on the flat surface and the PANI hierarchical structure showed a reduction of 1.6 log CFU and 0.60 log CFU, respectively (Figure 7c).

In contrast, pVD@PANI achieved a significant reduction of 3.0 log CFU, corresponding to 99.90% antibacterial efficiency. The corresponding colony-counting images show distinct differences among the control, pVD, PANI, and pVD@PANI (Figure 7b). This observation demonstrates that the hierarchical structure of PANI enhances the chemically induced antibacterial effect of a polymer coating.

## 4. Conclusions

In this study, we investigated the effects of molar concentrations of monomer, oxidant, and acid on the formation of nano- to microscale PANI structures on an NPA and the resulting antibacterial performance. By varying these reaction conditions, the morphology of PANI could be controlled, as confirmed via SEM. The antibacterial performance of an as-prepared pVD@PANI/Au HNS was successfully confirmed using pathogenic *E. coli* O157:H7 as a realistic model. The optimal antibacterial hybrid composite materials (0.01 M aniline, 0.0067 M APS, and 1 M perchloric acid) were fabricated using the advantages and structural properties of organic–inorganic materials. In addition, the coating of ionic antibacterial polymers on the surface of PANI nanostructure via iCVD (pVD@PANI/Au HNS) allowed for improved antibacterial performance. Moreover, the antibacterial properties (99.90%) were maximized by using the iCVD process to identify the synergies of the nanotopology and the antibacterial coating. Our findings demonstrate the relationship between the nanotopography and antibacterial properties of PANI/Au HNS and explain how an iCVD coating can improve materials for use in daily/harsh conditions such as biomedical diagnostics.

## Figures and Tables

**Table 1:** Comparison of reaction products according to concentrations of aniline and acid.

	[Aniline] ≥ [Acid]	[Aniline] < [Acid]
**Oxidation of Monomer**	Aniline radical	Anilinium cation
**Re-aromatization**	Phenazine-like oligomer	Para-coupled oligomer
**pH**	Weak acid (terminated)	Strong acid (terminated
**Structure**	Planar phenazine-like structure → π-π stacking	Many interchain/intrachain → H-bonding, π-π interaction, van der Waals force, entanglement
**Shape**	Nanosheet	Nanofiber or nanogranule

## Data Availability

Not applicable.

## Supplementary Materials

The following are available online at https://www.mdpi.com/article/10.3390/nano11102716/s1: Figure S1: SEM images of (a) Si mold, (b) PUA nanopillars; Figure S2: SEM images of (a) PUNO nanopillars, (b) Ti-deposited PUNO nanopillars, (c) Au/Ti-deposited PUNO nanopillars and (d) PANI/Au nanopillars.
