# Supplementary material for: 3D Hierarchical Polyaniline–Metal Hybrid Nanopillars: Morphological Control and Its Antibacterial Application
Source: Nanomaterials (Basel). 2021 Oct 14;11(10):2716. doi: 10.3390/nano11102716 (PMC8540657; doi:10.3390/nano11102716)
Supplement: Supplementary file 1 [file nanomaterials-11-02716-s001.zip › nanomaterials-1396151-supplementary.pdf]

Supplementary Materials

3D Hierarchical Polyaniline–Metal Hybrid Nanopillars: Morphological Control and Its Antibacterial Application

Jueun Kim [1], Younseong Song [2], Hogi Kim [2], Nam-Ho Bae [1], Tae Jae Lee [1], Yoo Min Park [1], Seok Jae Lee [1], Sung Gap Im [2], Bong Gill Choi [3,*] and Kyoung G. Lee [1,*]

[1] Center for Nano Bio Development, National NanoFab Center (NNFC), 291 Daehak-ro, Yuseong-gu, Daejeon 34141, Korea; jekim@nnfc.re.kr (J.K.); nhbae@nnfc.re.kr (N.-H.B.); tjlee@nnfc.re.kr (T.J.L.); ympark@nnfc.re.kr (Y.M.P.); sjlee@nnfc.re.kr (S.J.L.)

[2] Department of Chemical and Biomolecular Engineering, Korea Advanced Institute of Science and Technology (KAIST), 291 Daehak-ro, Daejeon, 34141, Korea; ong12ong12@kaist.ac.kr (Y.S.); hokiepokie91@kaist.ac.kr (H.K.); sgim@kaist.ac.kr (S.G.I.)

[3] Department of Chemical Engineering, Kangwon National University, Samcheok 25913, Korea

* Correspondence: bgchoi@kangwon.ac.kr (B.G.C.); kglee@nnfc.re.kr (K.G.L.)

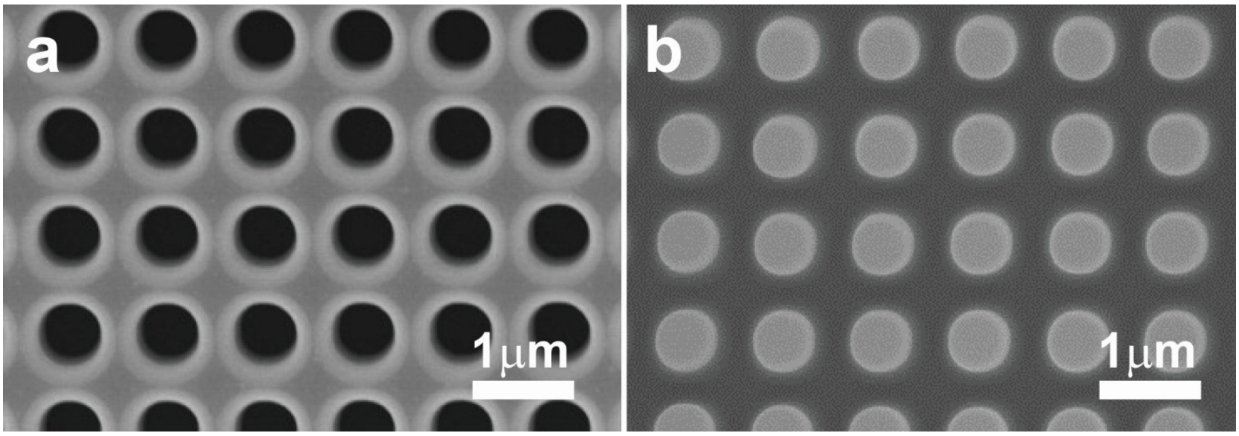

Figure S1. SEM images of (a) Si mold, (b) PUNO nanopillars.

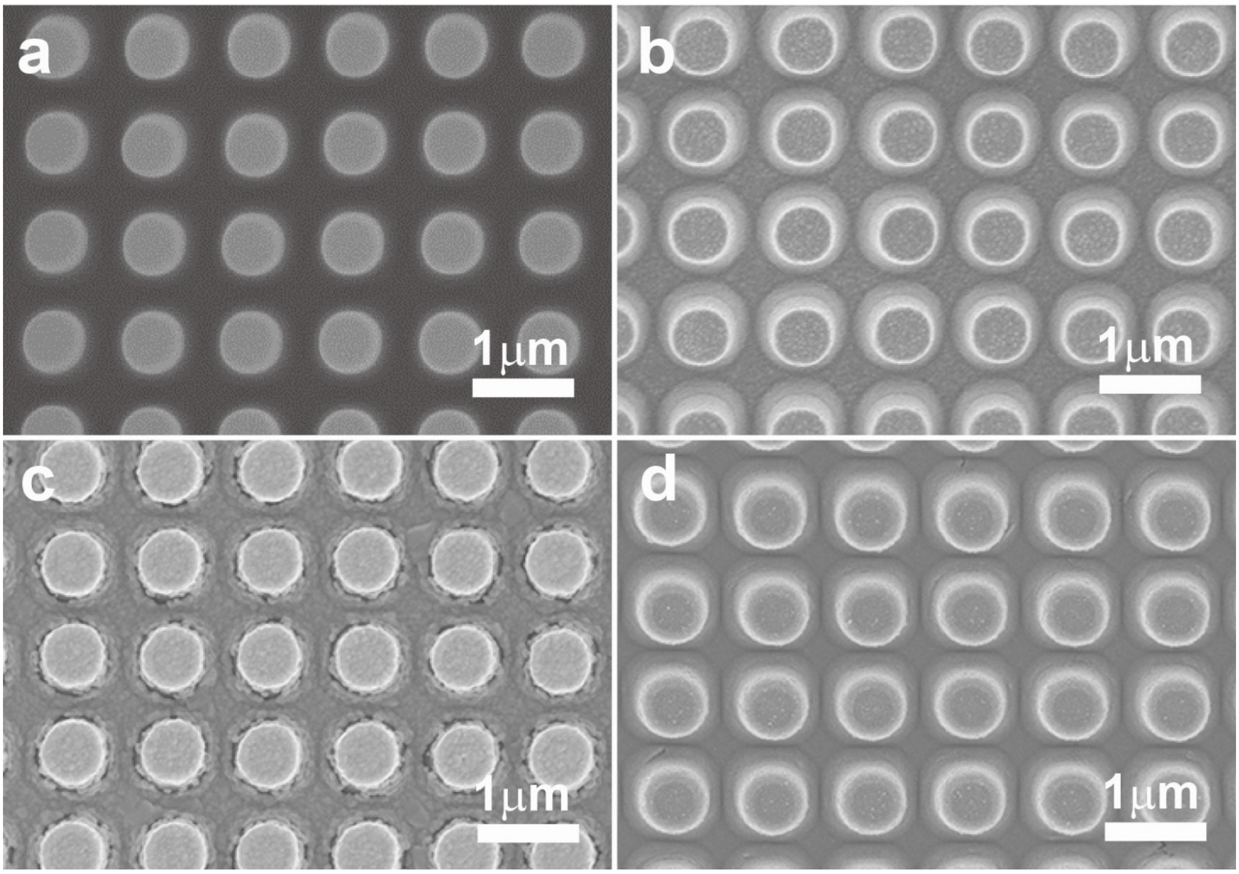

Figure S2. SEM images of (a) PUNO nanopillars, (b) Ti-deposited PUNO nanopollars, (c) Au/Ti-deposited PUNO nanopillars and (d) PANI/Au nanopillars.
